# Clustering cancer gene expression data: a comparative study

**DOI:** 10.1186/1471-2105-9-497

**Published:** 2008-11-27

**Authors:** Marcilio CP de Souto, Ivan G Costa, Daniel SA de Araujo, Teresa B Ludermir, Alexander Schliep

**Affiliations:** 1 Computational Molecular Biology, Max Planck Institute for Molecular Genetics, Berlin, Germany; 2 Dept. of Informatics and Applied Mathematics, Fed. Univ. of Rio Grande do Norte, Natal, Brazil; 3 Center of Informatics, Federal University of Pernambuco, Recife, Brazil

## Abstract

**Background:**

The use of clustering methods for the discovery of cancer subtypes has drawn a great deal of attention in the scientific community. While bioinformaticians have proposed new clustering methods that take advantage of characteristics of the gene expression data, the medical community has a preference for using "classic" clustering methods. There have been no studies thus far performing a large-scale evaluation of different clustering methods in this context.

**Results/Conclusion:**

We present the first large-scale analysis of seven different clustering methods and four proximity measures for the analysis of 35 cancer gene expression data sets. Our results reveal that the finite mixture of Gaussians, followed closely by *k*-means, exhibited the best performance in terms of recovering the true structure of the data sets. These methods also exhibited, on average, the smallest difference between the actual number of classes in the data sets and the best number of clusters as indicated by our validation criteria. Furthermore, hierarchical methods, which have been widely used by the medical community, exhibited a poorer recovery performance than that of the other methods evaluated. Moreover, as a stable basis for the assessment and comparison of different clustering methods for cancer gene expression data, this study provides a common group of data sets (benchmark data sets) to be shared among researchers and used for comparisons with new methods. The data sets analyzed in this study are available at .

## Background

Microarray technologies enable the measurement of molecular signatures of cancer cells. These data allow different types of analyses, such as (1) the identification of differentially expressed genes [1], which could indicate possible gene targets for more detailed molecular studies or drug treatments and (2) the building of classifiers, with machine learning techniques, which could be used to improve the diagnosis of patients with cancer [2]. Another common and more exploratory analysis is to perform a clustering of the cancer/patient samples (tissues). The aim is to find groups of samples sharing similar expression patterns, which can lead to the discovery of new cancer subtypes. This kind of analysis was first employed in [3] and [4]. Since then, clustering methods have drawn a great deal of attention in the scientific community [5]. Bioinformaticians have been proposing novel clustering methods that take intrinsic characteristics of gene expression data into account, such as noise and high-dimensionality, to improve the clusters [6-8]. Such researchers often perform an evaluation of their methods using available public data previously published in clinical studies. However, despite the relevant contributions provided by the bioinformatics community to the cluster analysis of microarray data, in order to aid the investigation of their data, researchers of clinical cancer studies still rely mostly on classic clustering methods, such as hierarchical clustering. Indeed, in studies using cluster analysis, the data of which are employed in this paper, around 95% are based on hierarchical clustering and Pearson's correlation as similarity metrics (e.g., [3,4,9-20]). The main reasons for such popularity of hierarchical clustering include (1) ease of use, as they require the setting of few parameters; and (2) the availability of implementations, as these methods are part of many standard microarray data analysis software programs, such as those in [21] and [22]. Hierarchical clustering is also available in standard gene expression databases, such as the Gene Expression Omnibus [23]. In contrast, common limitations of new methods proposed by bioinformaticians include the requirement of using particular programming environments and the specification of a number of different parameters (e.g., [6-8]), which makes their implementation difficult for non-expert users.

Motivated by these problems, we present the first large-scale analysis of different clustering methods and proximity measures for clustering cancer tissues (samples). The data employed were generated from gene expression measurement technologies. The aim is to provide some guidelines for the biological/medical/clinical community for the choice of specific methods. The study is based on 35 data sets from either Affymetrix or cDNA chip platforms. We evaluate the use of classic clustering methods, such as hierarchical clustering with single, complete and average linkage [24], *k*-means [25] and mixture of multivariate Gaussians [26]; as well as more recent methods, such as spectral clustering [27] and a nearest neighbor-based method [28]. Furthermore, in terms of proximity measures, we evaluate the choice of Pearson's correlation coefficient, cosine, Spearman's correlation coefficient and Euclidean distance [24]. For the case of Euclidean distance, we employ three procedures for pre-processing the expression profiles: standardization, scaling and ranking [29,30].

Before putting our analysis into perspective with respect to some of the related works, in order to prevent misleading interpretations, it is important to draw the attention to the fact that the problem of clustering cancer gene expression data (tissues) is very different from that of clustering genes. In the former, one has only tens or hundreds of items (tissues) to be clustered. Each of these data items is described by thousands of genes [2]. In contrast, in the task of clustering genes there are a large number of data items (genes), described by a small number of different conditions [5]. Thus, clustering few high dimensional items (tissues) is not the same as clustering several low dimensional ones (genes).

In either case, there are a small number of analyses in the literature evaluating the performance of different clustering methods applied to gene expression data [31-37]. In [37] the authors present basically a literature review of existing results on the use of several clustering algorithms in the context gene expression data. In terms of works that do develop an experimental analysis, [31-34,36] investigate the problem of clustering genes, differently from our study that has as a main concern the clustering of tissues. In fact, the most closely related work to ours is the one in [35]. There, among other things, the authors approach the problem of clustering cancer gene expression data sets. They perform their study in the context of four clustering methods (*k*-means, mixture of Gaussians, density-based clustering and farthest first traversal algorithm) applied to eight binary class data sets. Based on their experimental results obtained with the clustering methods, the authors do not suggest or indicate the suitability/preference of any of these methods with respect to the others. In contrast to the work in [35], as previously pointed out, our paper presents a first large scale analysis of seven different clustering methods and four proximity measures for the analysis of 35 cancer gene expression data sets. Furthermore, our data sets are not restricted to have only two classes.

Besides the large-scale comparison of clustering methods and proximity measures for cancer gene expression data, a major contribution of this paper is to provide a common group of data sets (benchmark data sets) to be shared among researchers as a stable basis for the evaluation and comparison of different machine learning methods for clustering or classification of cancer gene expression data – available in the supplementary material [38].

## Results and discussion

### Experimental Design

Thirty five publicly available microarray data sets are included in our analysis (Table 1). These data sets were obtained using two microarrays technologies: single-channel Affymetrix chips (21 sets) and double-channel cDNA (14 sets). We compare seven different types of clustering algorithms: single linkage (SL), complete linkage (CL), average linkage (AL), *k*-means (KM), mixture of multivariate Gaussians (FMG), spectral clustering (SPC) and shared nearest neighbor-based clustering (SNN). When applicable, we use four proximity measures together with these methods: Pearson's Correlation coefficient (P), Cosine (C), Spearman's correlation coefficient (SP) and Euclidean Distance (E). Regarding Euclidean distance, we employ the data in four different versions: original (*Z*_0_), standardized (*Z*_1_), scaled (*Z*_2_) and ranked (*Z*_3_) versions. See the Methods Section for further details on the algorithms, proximity measures and data transformation procedures.

**Table 1:** Data set description

**Dataset**	**Chip**	**Tissue**	***n***	**#C**	**Dist. Classes**	***m***	***d***
Armstrong-V1 [52]	Affy	Blood	72	2	24,48	12582	1081
Armstrong-V2 [52]	Affy	Blood	72	3	24,20,28	12582	2194
Bhattacharjee [9]	Affy	Lung	203	5	139,17,6,21,20	12600	1543
Chowdary [13]	Affy	Breast, Colon	104	2	62,42	22283	182
Dyrskjot [14]	Affy	Bladder	40	3	9,20,11	7129	1203
Golub-V1 [3]	Affy	Bone marrow	72	2	47,25	7129	1877
Golub-V2 [3]	Affy	Bone marrow	72	3	38,9,25	7129	1877
Gordon [53]	Affy	Lung	181	2	31,150	12533	1626
Laiho [15]	Affy	Colon	37	2	8,29	22883	2202
Nutt-V1 [54]	Affy	Brain	50	4	14,7,14,15	12625	1377
Nutt-V2 [54]	Affy	Brain	28	2	14,14	12625	1070
Nutt-V3 [54]	Affy	Brain	22	2	7,15	12625	1152
Pomeroy-V1 [55]	Affy	Brain	34	2	25,9	7129	857
Pomeroy-V2 [55]	Affy	Brain	42	5	10,10,10,4,8	7129	1379
Ramaswamy [50]	Affy	Multi-tissue	190	14	11,10,11,11,22,10,11,10,30,11,11,11,11,20	16063	1363
Shipp [56]	Affy	Blood	77	2	58,19	7129	798
Singh [19]	Affy	Prostate	102	2	58,19	12600	339
Su [57]	Affy	Multi-tissue	174	10	26,8,26,23,12,11,7,27,6,28	12533	1571
West [58]	Affy	Breast	49	2	25,24	7129	1198
Yeoh-V1 [20]	Affy	Bone marrow	248	2	43,205	12625	2526
Yeoh-V2 [20]	Affy	Bone marrow	248	6	15,27,64,20,79,43	12625	2526
Alizadeh-V1 [4]	cDNA	Blood	42	2	21,21	4022	1095
Alizadeh-V2 [4]	cDNA	Blood	62	3	42,9,11	4022	2093
Alizadeh-V3 [4]	cDNA	Blood	62	4	21,21,9,11	4022	2093
Bittner [10]	cDNA	Skin	38	2	19, 19	8067	2201
Bredel [11]	cDNA	Brain	50	3	31,14,5	41472	1739
Chen [12]	cDNA	Liver	180	2	104,76	22699	85
Garber [59]	cDNA	Lung	66	4	17,40,4,5	24192	4553
Khan [60]	cDNA	Multi-tissue	83	4	29,11,18,25	6567	1069
Lapointe-V1 [16]	cDNA	Prostate	69	3	11,39,19	42640	1625
Lapoint-V2 [16]	cDNA	Prostate	110	4	11,39,19,41	42640	2496
Liang [17]	cDNA	Brain	37	3	28,6,3	24192	1411
Risinger [18]	cDNA	Endometrium	42	4	13,3,19,7	8872	1771
Tomlins-V1 [61]	cDNA	Prostate	104	5	27,20,32,13,12	20000	2315
Tomlins-V2 [61]	cDNA	Prostate	92	4	27,20,32,13	20000	1288

The data sets present different values for features such as type of microarray chip (second column), tissue type (third column), number of samples (fourth column), number of classes (fifth column), distribution of samples within the classes (sixth column), dimensionality (seventh column) and dimensionality after feature selection (last column).

**Figure 1:**
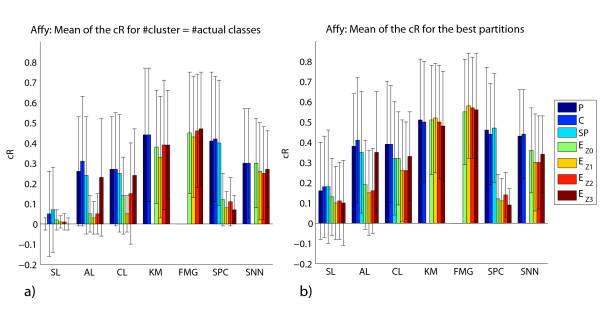
**Affymetrix data sets: mean of the cR**. We display the mean of the cR for the partitions with the number of clusters equal to the actual number of classes (a) and the mean of the best cR found (b) for Affymetrix data sets. Missing bars correspond to combinations of methods and proximity measures not evaluated.

**Figure 2:**
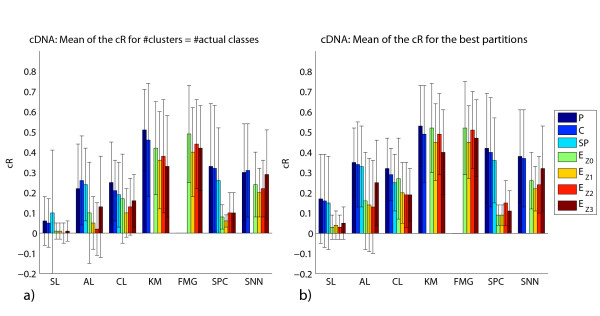
**cDNA data sets: mean of the cR**. We display the mean of the cR for the partitions with the number of clusters equal to the actual number of classes (a) and the mean of the best cR found (b) for cDNA data sets. Missing bars correspond to combinations of methods and proximity measures not evaluated.

**Figure 3:**
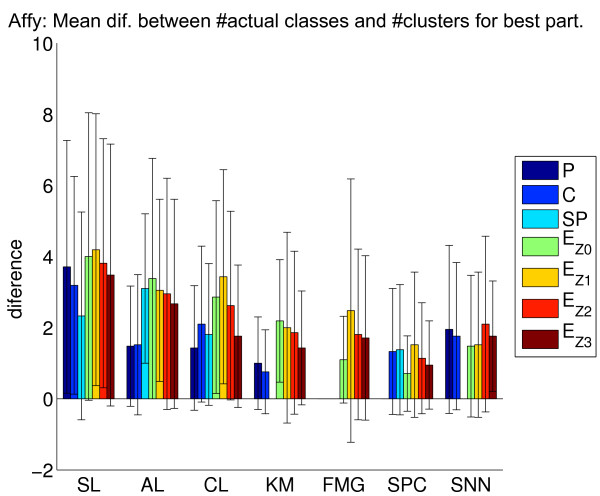
**Affymetrix data sets: difference between the actual number of classes and the number of clusters in the partition solutions with best cR**. We display the mean of the difference between the actual number of classes and the number of clusters for the best partition found for Affymetrix data sets. Missing bars correspond to combinations of methods and proximity measures not evaluated.

**Figure 4:**
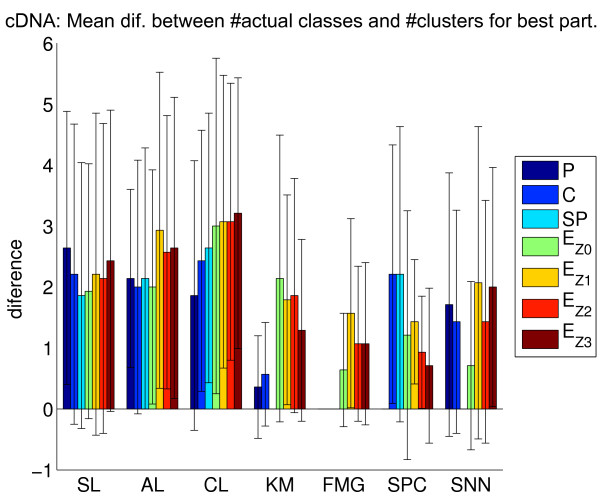
**cDNA data sets: difference between the actual number of classes and the number of clusters in the partition solutions with best cR**. We display the mean of the difference between the actual number of classes and the number of clusters for the best partition found for cDNA data sets. Missing bars correspond to combinations of methods and proximity measures not evaluated.

We perform experiments for each algorithm, varying the number of clusters in [*k*, ⌈$\sqrt{n}$⌉], where *k *represents the actual number of classes in a given data set with *n *samples. The recovery of the cluster structure is measured using the corrected Rand (cR) index by comparing the actual classes of the tissues samples (e.g., cancer types/subtypes) with the cluster assignments of the tissue samples. For each combination of clustering method, proximity measure and data transformation procedure, we calculate the mean of the cR over all data sets in two different contexts: (1) taking into account only the partition with the number of clusters equal to the number of actual classes in the respective data set; and (2) considering the partition that presents the best cR for each data set, regardless of its number of clusters. The results of these experiments are illustrated in Figure 1 (Affymetrix data sets) and Figure 2 (cDNA data sets). We also investigate the influence of reduced coverage on the algorithms [29]. The idea is to identify the methods that often generate their best partition, as calculated by the cR, with more clusters than the actual number of classes in the corresponding data set. In order to do so, for a given algorithm, we measure the difference between the number of clusters of the partition with best cR and the actual number of classes for each data set. The mean of the difference of these values for Affymetrix and cDNAs data sets are displayed in Figures 3 and 4, respectively.

Finally, we perform paired *t*-tests, adjusted for multiple comparisons with the Benjamini and Hochberg procedure [39], to determine whether differences in the cR between distinct methods (as displayed in Figure 1 and Figure 2) are statistically significant. More specifically, given that we are mainly interested in comparing the clustering methods, we select only the results with the proximity measure that displays the largest mean of cR for each method. As some data sets are harder to cluster than others, the cR values exhibited a large variance for a given clustering method and proximity measure (Figures 1 and Figure 2). Nonetheless, as all methods are applied to the same data sets, we can use the paired *t*-test, which measures the difference in the cR value between two methods for each data set. In this case, the null hypothesis is that the difference in the mean between paired observations is zero. We can therefore assess whether the difference in the performance of one method and another is statistically significant. More detailed results are found out in our supplementary material [38].

### Recovery of Cancer Types by Clustering Method

Based on Figures 1 and 2, our results show that, among the 35 data sets investigated, the FMG exhibited the best performance – best corrected Rand (cR) -, followed closely by KM, in terms of the recovery of the actual structure of the data sets, regardless of the proximity measure used.

For Affymetrix data sets, the paired *t*-test (*p*-value < 0.05) indicated that FMG achieved a larger cR than SL, AL and CL; and KM a larger cR than SL and AL, when the number of cluster is set to the actual number of classes. Considering the partition that presents the best cR for each data set, regardless of its number of clusters, KM and FMG achieved a larger cR than SL, AL and CL (*p*-value < 0.05).

In the case of cDNA data sets, the paired *t*-test (*p*-value < 0.05) also indicated that KM and FMG achieved a larger cR than SL, AL, CL and SNN for both contexts investigated. Also, KM achieved a larger cR than SPC (*p*-value < 0.05). This holds for both contexts: the number of cluster sets to the actual number of classes and for the best cR, regardless of the number of clusters.

Furthermore, KM and FMG achieved, on average, the smallest difference between the actual number of classes in the data sets and the number of clusters in the partition solutions with the best cR (Figures 3 and 4). Likewise, SNN exhibited consistent behavior in terms of the values of cR in the different types of proximity measures, although with smaller cR values than those obtained with FMG and KM. In fact, this method, on average, returned cR values compatible to those achieved by the SPC.

Note that a good coverage alone (Figure 3 and Figure 4) do not imply accuracy in classes recovery (Figures 1 and 2). For example, according to Figure 3, SPC with ${\text{E}}_{Z_{0}}$ and KM with C respectively present a mean of the difference between the actual number of classes and number of clusters found in the partitions with best cR of 0.71 and 0.76 clusters. However, the latter led to a cR of 0.50, while the former achieved a cR of only 0.12.

Our results show that the class of hierarchical methods, on average, exhibited a poorer recovery performance than that of the other methods evaluated. Moreover, as expected, within this class of algorithms, the single linkage achieved the worst results. The paired *t*-test (*p*-value < 0.05) indicated that it led to the smallest cR, when compared to those of the other methods. Regarding the use of proximity measures with hierarchical methods, Pearson's correlation and cosine yielded best results. This is also in agreement with the overall common knowledge from clinical/biological studies [22]. In order to present cR values compatible to those obtained with KM and FMG, such a class of methods generally required a much more reduced coverage: a larger number of clusters than that in the underlying data. For example, according to Figure 1, the AL with P, achieves a cR of 0.27 for the actual number of classes. In contrast, if one considers partition solutions with a larger number of clusters, the cR increases to 0.38. Such an increase was achieved with partitions that had, on average, 1.48 more clusters than in the underlying structure. One surprising result is the good performance achieved with *Z*_3_, mainly with the hierarchical clustering methods. In this context, the use of such a data transformation, together with the Euclidean distance, led to results very close to those obtained with P, C and SP, especially for the Affymetrix data sets. One reason for this behavior is the presence of outliers in the data sets, as *Z*_3 _reduces their impact [29]. Thus, this is further evidence that such a class of algorithms is more susceptible to outliers than the other methods investigated.

Spectral clustering, in turn, is quite sensitive to the proximity measure employed. For example, the partitions generated with this method achieved large cR values (e.g., cR > 0.40) only for the cases of C, SP and P, but smaller cR values (e.g., cR ≤ 0.15) otherwise. It is well known in the literature that spectral clustering is susceptible to the similarity matrix used [40]. Moreover, there is as yet no rule to assist in making such a selection.

In another kind of analysis, we investigated the impact of reduced coverage on the performance of the algorithms. As previously mentioned, this impact was more significant for the case of the hierarchical clustering methods. As there are many data sets with an extremely unbalanced distribution of samples within the classes, this behavior also occurred with the KM, although to a smaller degree. In fact, all methods, even the ones that are supposed to deal well with clusters with an unbalanced number of samples, profited from a reduced coverage.

### Comparison of Hierarchical Clustering and *k*-means

To better illustrate the issues discussed in the previous section, we analyzed the results obtained with *k*-means and hierarchical clustering for a single data set. More specifically, we employed a version of the data set in [4] (Alizadeth-V2), which has samples from three cancer types: 42 diffuse large B-cell lymphoma (DLBCL), 9 follicular lymphoma (FL) and 11 chronic lymphocytic leukemia (CLL). In the case of hierarchical clustering, we used the same algorithm as in [4], which is hierarchical clustering with average linkage and Pearson's correlation. In order to improve visualization of the red and green plot [22], the leaves of the resulting tree were rearranged according to a standard technique [41].

**Figure 5:**
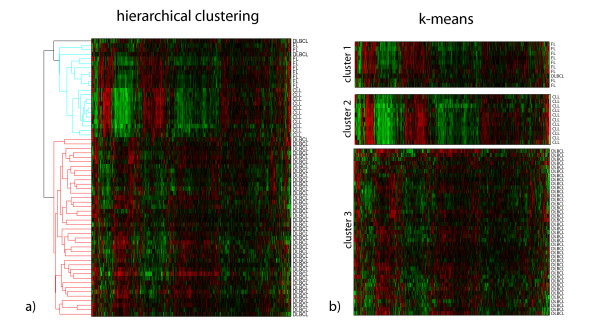
**Hierarchical Clustering and *k*-means for Alizadeh-V2**. We display the red and green plots for (a) the hierarchical clustering and (b) the *k*-means for the data set Alizadeh-V2. Columns correspond to genes and rows correspond to cancer samples. The samples are labeled according to one of three classes: diffuse large B-cell lymphoma (DLBCL), follicular lymphoma (FL) and chronic lymphocytic leukemia (CLL). In the case of *k*-means, the number of clusters was set at three. Likewise, for hierarchical clustering, the tree was cut so as to return three clusters, corresponding to the red, light blue and black sub-trees.

The partitions with three clusters for hierarchical clustering and *k*-means are illustrated in Figure 5(a) and Figure 5(b), respectively. The *k*-means yielded a partition very close to the underlying structure in the data. With the exception of a single DLBCL sample that was wrongly assigned to Cluster 1 (all other samples in this cluster are FL), the other clusters in the partition have samples of only the given class. In the case of hierarchical clustering, cutting the tree at level three (three sub-trees or clusters), one can observe the following: the red sub-tree contains only DLBCL samples and the blue sub-tree presents a combination of FL and CLL samples. The same DLBCL sample wrongly assigned in the case of *k*-means appeared in the blue sub-tree rather than the red sub-tree. The third sub-tree (black) is formed by a single sample from DLBCL (top). Indeed, in the tree generated, the FL and CLL samples are only separated if we cut the tree at level 25, which yields 25 sub-trees (clusters). Note that, although our tree differs slightly from the one in [4], as their analysis included additional non-cancer tissue samples; in that paper the FL and CLL samples would also only be in separate sub-trees when the tree is cut at a lower level.

**Figure 6:**
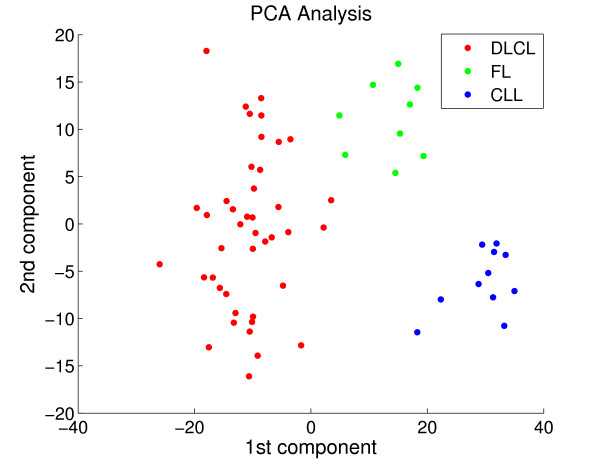
**PCA plot for Alizadeh-V2**. We display a scatter plot with the two first largest components of a PCA for Alizadeh-V2. Colors indicate the three classes in the data: diffuse large B-cell lymphoma in red (DLBCL), follicular lymphoma in green (FL) and chronic lymphocytic leukemia in blue(CLL).

One of the reasons for this kind of problem is that hierarchical clustering is based on local decisions, merging the most "compact" cluster available at each step [24]. The compactness criterion (or how close together objects are) is defined by the linkage criteria and proximity measure used. On the other hand, *k*-means maximizes a criterion, which is a combination of cluster compactness and cluster separation. The problem can be illustrated in a two-dimensional representation of the Alizadeth-V2 data set, after selecting the two-largest components using principal component analysis (PCA) – Figure 6. Based on this figure, we can see that samples from distinct classes form natural clusters. More specifically, although the cluster with DLBCL samples (red dots) is well-separated from the other two clusters, it lies within a non-compacted region. Thus, as hierarchical clustering has a bias towards compacted clusters, if we simply follow the hierarchical tree, it would first suggest the sub-division of the cluster with DLBCL samples before sub-dividing the groups with FL and CLL samples. In a hypothetical scenario where there is no a priori knowledge on the distinction between FL and CLL samples, the use of hierarchical clustering alone would not indicate the existence of these two classes. In contrast, this would be detected with *k*-means or clearly visualized with a simple PCA analysis.

## Conclusion

We have provided the first large-scale data-driven comparative study of seven clustering algorithms and four proximity measures applied to 35 cancer gene expression data sets. In the following, we summarize some of the general trends (guidelines for clustering cancer gene expression data) that emerged from our comparative study.

1. Overall, among the 35 data sets investigated, the FMG exhibited the best performance, followed closely by KM, in terms of the recovery of the actual structure of the data sets, regardless of the proximity measure used.

2. For most algorithms, there is a clear interaction between reduced coverage and an increase in the ability of the algorithm to group the samples correctly – larger corrected Rand.

3. The shortcomings of hierarchical methods is noticeable, as it has been the case in the analyses developed in the context of clustering genes [31,32]. One of the reasons for this is the sensitivity of hierarchical clustering to noise in the data [5,24,29].

4. Within this class of hierarchical clustering algorithms, the single linkage presented the worst results.

5. With respect to the use of proximity measures with hierarchical clustering methods, Pearson's correlation and cosine often led to the best results.

6. To present cR values compatible to those obtained with KM and FMG, the class of hierarchical clustering methods usually required a much more reduced coverage.

7. Spectral clustering showed to be quite sensitive to the proximity measure employed.

With respect to the statement in Item 1, it is important to point out that, although, on average, our experimental work demonstrates that FMG and KM exhibited a better performance in terms of the corrected Rand than the other methods investigated, this does not imply that these algorithms would always be the best choice. Indeed, as one can see in the table in the supplemental material describing the individual performance of the algorithms, for certain data sets, such as Pomeroy-V2, the SNN with P achieved a much larger cR than all the other methods [38].

A principled way to tackle this problem of predicting which methods would work better for a certain data set with particular data properties (i.e., number of samples, sample dimensionality, array type, etc.) is the use of meta-learning approaches [42]. For example, in a smaller scale study, [43] shows that a meta-learning approach taking into consideration only a set of descriptive statistics of a given data as input, yields a ranking with the best clustering methods to be used with the data set.

Another contribution of this paper was that we provided a common group of data sets (benchmark data sets) that can be used as a stable basis for the evaluation and comparison of different machine learning methods. Recently, studies proposing benchmarking frameworks have been introduced in the machine learning and bioinformatics literature [44,45]. For instance, [44] has proposed a web-based system for the storage of results from classification methods for several benchmark data sets used in the machine learning community. Furthermore, their database allows other researchers to submit results obtained by new classification methods. In fact, as future work, we aim to build a central repository for the evaluation of clustering methods in the context of cancer gene expression data. In this repository, any novel clustering algorithm could be evaluated with the available data sets, its results stored and made available in our web database.

## Methods

### Clustering Methods and Recovery Measure

Seven clustering algorithms are used to generate partition solutions and form a single factor in the overall experiment design: single linkage, complete linkage, average linkage, *k*-means, mixture of multivariate Gaussians, spectral clustering and shared nearest neighbor-based clustering (SNN). These algorithms have been chosen to provide a wide range of recovery effectiveness, as well as to give some generality to the results. In our analysis, when applicable, methods are implemented with Pearson's Correlation, cosine and Euclidean distance. We also use single linkage, complete linkage, average linkage and spectral clustering with the Spearman's correlation coefficient – the implementation that we used for *k*-means (Matlab) and SNN [28] do not support such a proximity measure.

Hierarchical clustering methods, more specifically the agglomerative ones, are procedures for transforming a distance matrix into a dendrogram [24]. Such algorithms start with each sample representing a cluster. The methods then gradually merge these clusters into larger ones; they start with trivial clustering in which each sample is in a unique cluster, ending with trivial clustering in which all samples are in the same cluster. There are three more widely used variations of hierarchical clustering, which are used in this paper: complete linkage (CL), average linkage (AL) and single linkage (SL). These variations differ in the way the distance between two clusters is calculated. For SL, the distance between two clusters is determined by the two closest samples in different clusters. In contrast, CL employs the farthest distance of a pair of samples to define the inter-cluster distance. In AL, the distance between two clusters is calculated by the average distance between the samples in one group and the samples in the other group. This method has been used extensively in the literature on gene expression analysis [31,32,46,47], although experimental results have shown that, in many cases, the complete linkage outperforms it [5]. Another widely used method for gene expression data analysis is *k*-means [46,47]. *k*-means (KM) is a partitional iterative algorithm that optimizes the best fitting between clusters and their representation using a predefined number of clusters [24,25]. Starting with prototype values from randomly selected samples, the method works in two alternate steps: (1) an allocation step, where all samples are allocated to the cluster containing the prototype with the lowest dissimilarity; and (2) a representation step, where a prototype is constructed for each cluster. One problem with such an algorithm is its sensitivity to the selection of the initial partition. This could lead the algorithm to converge to a local minimum [24]. In order to prevent the local minimum problem, a number of runs with different initializations is often performed. The best run based on some cohesion measure is then taken as the result. Another characteristic of this method is its robustness to noisy data.

Finite mixture of Gaussians (FMG) is a convex summation of *k *multivariate Gaussian density functions or Gaussian components [26]. In mixture model-based clustering, each component in the mixture is assumed to model a group of samples. In order to obtain the probabilities of a sample belonging to each cluster (group), density functions can be used and mixing coefficients can be defined for the mixture. For a given number *k*, mixture models can be efficiently computed with the Expectation-Maximation (EM) algorithm. The EM works interactively by calculating the probability of each sample for the components and then recomputing the parameters of the individual Gaussian densities until convergence is reached. A mixture model has a number of advantages: it returns the uncertainty of a given cluster assignment and the models estimated can be seen as descriptors of the clusters found. In practice, *k*-means can be regarded as an oversimplification of a mixture of Gaussians, where every sample is assigned to a single cluster. In this simplified context, clusters tend to have the same size and lie within spherical regions of the Euclidean space. With the mixture of Gaussians using unit covariance matrices employed in this paper, clusters will also lie within spherical regions, but can have any arbitrary size.

Spectral clustering (SPC) is a general class of algorithms characterized by employing the spectrum of similarity matrices to reduce the dimensionality of a data set and then applying a basic clustering algorithm, such as *k*-means or graph cut-based methods, on this lower dimension data [27]. For example, assume that we have a graph, where nodes are the samples and edges are weighed by the similarity between two nodes. Spectral clustering methods can be interpreted as performing a random walk in this graph, finding clusters by ignoring edges that are rarely transversed in the walk. More specifically, in this paper, for a given similarity matrix *S *obtained with a Gaussian similarity function, we (1) calculate its normalized Laplacian matrix and (2) perform an eigenvalue decomposition of this matrix. We then select the *k *eigenvectors related to the *k *lowest eigenvalues and use them to perform *k*-mean clustering. Among other interesting characteristics, a spectral clustering method makes no assumptions on the data distribution at hand. It is also able to find clusters that are not in convex regions of the space.

The shared nearest-neighbor algorithm (SNN) is a recent technique. We have added it to our analysis because this method can robustly deal with high dimensionality, noise and outliers [28]. SNN searches for the nearest neighbors of each sample and uses the number of neighbors that two points share as the proximity index between them. With this index, SNN employs a density-based approach to find representative samples and build clusters around them. Along with the number of nearest neighbors (*NN*), two other kinds of parameters are considered: those regarding the weights of the shared nearest neighbor graph (strong, merge and label) and others related to the number of strong links (topic and noise). These parameters are thresholds on which each step of the algorithm is based.

Regarding the index for measuring the success of the algorithm in recovering the true partition of the data sets, as in [29], we use the corrected Rand [24,29]. The corrected Rand index takes values from -1 to 1, with 1 indicating a perfect agreement between the partitions and values near 0 or negatives corresponding to cluster agreement found by chance. Unlike the majority of other indices, the corrected Rand is not biased towards a given algorithm or number of clusters in the partition [24,48].

Formally, let *U *= {*u*_1_,...,*u*_*r*_,...,*u*_*R*_} be the partition given by the clustering solution and *V *= {*v*_1_,...,*v*_*c*_,...,*v*_*C*_} be the partition formed by a priori information independent from partition U (thegold standard). The corrected Rand is defined as:

$$cR=\frac{\sum_{i}^{R}\sum_{j}^{C}\left(\begin{array}{c} n_{ij}\\ 2 \end{array}\right)-{\left(\begin{array}{c} n\\ 2 \end{array}\right)}^{-1}\sum_{i}^{R}\left(\begin{array}{c} n_{i}\\ 2 \end{array}\right)\sum_{j}^{C}\left(\begin{array}{c} n._{j}\\ 2 \end{array}\right)}{\frac{1}{2}\left[\sum_{i}^{R}\left(\begin{array}{c} n_{i}.\\ 2 \end{array}\right)+\sum_{j}^{C}\left(\begin{array}{c} n._{j}\\ 2 \end{array}\right)\right]-{\left(\begin{array}{c} n\\ 2 \end{array}\right)}^{-1}\sum_{i}^{R}\left(\begin{array}{c} n_{i}.\\ 2 \end{array}\right)\sum_{j}^{C}\left(\begin{array}{c} n._{j}\\ 2 \end{array}\right)}$$

where (1) *n*_*ij *_represents the number of objects in clusters *u*_*i *_and *v*_*j*_; (2) *n*_*i*. _indicates the number of objects in cluster *u*_*i*_; (3) *n*._*j *_indicates the number of objects in cluster *v*_*j*_; (4) *n *is the total number of objects; and(5) $\left(\begin{array}{c} a\\ b \end{array}\right)$ is the binomial coefficient $\frac{a!}{b!(a-b)!}$.

### Data Transformation

In many practical situations, a data set could present samples the attribute or feature values of which (in our case, genes) lie within different dynamic ranges [24,29]. In this case, for proximity measures such as Euclidean distance, features with large values will have a greater influence than those with small values. However, this will not necessarily reflect their importance in defining the clusters. Such a problem is often addressed by transforming the feature values so that they lie within similar ranges. There are several ways to perform transformations of attribute values [24,29]. As the data sets used in our studies have no categorical features, we consider only the case involving numeric values. More precisely, for the Euclidean distance, we analyze three different forms of feature (gene) transformation.

The first two have been widely used in clustering applications [24,29]: one is based on the *z*-score formula (standardization) and the other scales the gene values to [0, 1] or [-1, 1]. The third procedure transforms the values of the attributes into a ranking. This type of transformation is more robust to outliers than the first two procedures [29]. A large-scale study on the impact of these procedures for cancer gene expression data has been recently presented in [30].

The transformation that uses the *z*-score formula translates and scales the axes so that transformed feature (gene) will have zero mean and unit variance. Hereafter, for short, we will refer to this transformation as *Z*_1_. The second procedure involves the use of the maximum and minimum values on the gene. Assuming non-negative values, a gene transformed with this procedure is bounded by 0.0 and 1.0, with at least one observed value at each of these end points [29]. For short, we will refer to this procedure as *Z*_2_. If there are negative values, a gene transformed with *Z*_2 _is bounded by -1.0 and 1.0. Unlike *Z*_1_, the transformed mean and variance resulting from *Z*_2 _will not be constant across all features.

The above procedures could be adversely affected by the presence of outliers in the genes. This is especially true for *Z*_2_, which depends on the minimum and maximum values. A different approach, which is more robust to outliers, converts the values of the genes to rankings. Given a data set with *n *samples, this procedure yields a transformed gene value with mean of (*n *+ 1)/2, range *n *- 1 and variance (*n *+ 1) [((2*n *+ 1)/6) - ((*n *+ 1)/4)] for all genes [29]. For short, hereafter, we will refer to this procedure as *Z*_3_.

### Experimental Design

#### Data sets

Thirty five microarray data sets are included in our analysis (Table 1). These data sets present different values for features such as type of microarray chip (second column), tissue type (third column), number of samples (fourth column), number of classes (fifth column), distribution of samples within the classes (sixth column), dimensionality (seventh column) and dimensionality after feature selection (last column).

In terms of the data sets, it is important to point out that microarray technology is generally available in two different types of platforms, single-channel microarrays (e.g., Affymetrix) or double-channel microarrays (e.g., cDNA) [46,47,49]. Note that other microarrays technologies are also based on either single and double channels methods. As the data sets analyzed here are restricted to those collected with cDNA and Affymetrix microarrays, we employ the terms cDNA and Affymetrix to denote double or single-channel arrays, respectively. Measurements of Affymetrix arrays are estimates on the number of RNA copies found in the cell sample, whereas cDNA microarrays values are ratios of the number of copies in relation to a control cell sample. With Affymetrix microarray data, following other works, all genes with expression level below 10 are set to a minimum threshold of 10. The maximum threshold is set at 16,000. Values below or above these thresholds are often unreliable [49-51]. Thus, our analysis is performed on the scaled data to which the ceiling and threshold values have been applied.

Moreover, for the case of Affymetrix data, we apply the following procedure to remove uninformative genes: for each gene *j *(attribute), we compute the mean *m*_*j *_among the samples. However, in order to get rid of extreme values, we first discard the 10% largest and smallest values. Based on this mean, we transform every value $x_{ij}^{*}$ of gene *i *and sample *j *as follows:

$$y_{ij}=\log_{2}\left(x_{ij}^{*}/m_{j}\right).$$

For further analysis, we then select genes with expression levels differing by at least *l*-fold in at least *c *samples from their mean expression level across the samples. With few exceptions, the parameters *l *and *c *were chosen so as to produce a filtered data set with at least 10% of the original number of genes (features). It is important to point out that the data transformed with the previous equation is only used in the filtering step.

A similar filter procedure was applied for the case of cDNA microarray, but without the need to transform the data. In the case of cDNA microarray data sets, attributes (genes) of which could present missing values, we discard those with more than 10% of missing values. The attributes that remain and still present missing values have the values replaced by the respective mean value of the attribute.

### Empirical Study

The experiments compare seven different types of clustering algorithms: SL, AL, CL, KM, FMG, SPC and SNN. Each of these algorithms (with the exception of FMG, which already has the computation of the similarity as a built-in characteristic) was implemented with versions of four proximity measures: Pearson's Correlation coefficient (P), Cosine (C), Spearman's correlation coefficient (SP) and Euclidean Distance (E). As the implementation of the KM and SNN used in this work does not support Spearman's correlation coefficient, these algorithms were only tested with P, C and E. Furthermore, regarding the Euclidean distance version, experiments are performed with the samples in four forms, namely, original (*Z*_0_), standardized (*Z*_1_), normalized (*Z*_2_) and ranked (*Z*_3_). We also applied the data with these transformations for the case of FMG.

We run the algorithms for the configurations described in the previous paragraph. Recovery of cluster structure was measured via the corrected Rand index with regard to the actual number of classes known to exist in the data. That is, the number of clusters is set to be equal to the true number of the classes in the data. The known class labels were not used in any way during the clustering. As performed by [29], in order to explore the effects of reduced coverage, recovery was also measured on different levels preceding the correct solution partition. Reduced coverage implies that more clusters are present in the partition obtained than actually exist in the underlying structure. For example, if *k *represents the number of classes in a data set with *n *samples, we then developed experiments varying the number of clusters in [*k*, ⌈$\sqrt{n}$⌉]. In order to build the partition from hierarchical methods, recovery was measured based on the hierarchical solution that corresponds to each value in the range [*k*, ⌈$\sqrt{n}$⌉]. As KM is non-deterministic, we run the algorithm 30 times for each configuration pair (data set, proximity measure) and select the one with lowest inter-class error. For further analysis, we then choose the partition with the best corrected Rand (cR). This procedure is also used for SPC and FMG. We use default parameters in both cases, with the exception of the values for the number of clusters.

For SNN, we execute the algorithm with several values for its parameters (2%, 5%, 10%, 20%, 30% and 40% for *NN*), topic (0, 0.2, 0.4, 0.6, 0.8 and 1) and merge (0, 0.2, 0.4, 0.6, 0.8 and 1). Preliminary experiments reveal that variations in the other parameters did not produce very different results. Thus, the default values were used for the parameter strong and the value 0 was used for the parameters noise and label (in order to have all points assigned to a cluster). From the partitions created with such parameters values, we choose the partition with best cR and with *k *in the interval of interest for further analysis.

## Authors' contributions

DA implemented the approach and performed the experiments. DA, MS and IC evaluated the results. MS, IC, TL and AS designed the study and wrote the manuscript. All authors read and approved the final manuscript.
